# Introspection about backward crosstalk in dual-task performance

**DOI:** 10.1007/s00426-019-01282-3

**Published:** 2020-01-23

**Authors:** Daniel Bratzke, Markus Janczyk

**Affiliations:** 1 Department of Psychology, Eberhard Karls University of Tübingen, Tübingen, Germany; 2 Department of Psychology, University of Bremen, Bremen, Germany

## Abstract

The present study investigated participants’ ability to introspect about the effect of between-task crosstalk in dual tasks. In two experiments, participants performed a compatibility-based backward crosstalk dual task, and additionally provided estimates of their RTs (introspective reaction times, IRTs) after each trial (Experiment 1) or after each pair of prime and test trials (Experiment 2). In both experiments, the objective performance showed the typical backward crosstalk effect and its sequential modulation depending on compatibility in the previous trial. Very similar patterns were observed in IRTs, despite the typical unawareness of the PRP effect. In sum, these results demonstrate the reliability of between-task crosstalk in dual tasks and that people’s introspection about the temporal processing demands in this complex dual-task situation is intriguingly accurate and severely limited at the same time.

## Introspection about backward crosstalk in dual-task performance

A long tradition of dual-task research has shown that multitasking usually comes at a cost (for reviews see Koch, Poljac, Müller, & Kiesel, 2018; Pashler, 1994). These costs are frequently investigated with psychological refractory period (PRP) experiments (e.g., Pashler, 1994), where two tasks (Task 1 and Task 2) are presented with varying temporal overlap (stimulus onset asynchrony, SOA). Typically, reaction time (RT) for Task 2 (RT2) increases with increasing overlap (i.e., with decreasing SOA), the so-called PRP effect (Telford, 1931). The prominent central bottleneck model attributes this effect to a resource-limited central processing stage, which can serve only one task at a time (Welford, 1952; Pashler, 1994). Accordingly, central processing in Task 2 cannot start until the central processor is freed from Task 1 central processing. At short SOAs, when there is a high temporal overlap between the two tasks, this leads to a waiting time in Task 2 processing and a corresponding prolongation of RT2.

Numerous studies have demonstrated that introspection about dual-task costs is severely limited, since participants are usually not aware of the PRP effect (Bratzke & Bryce, 2016; Bryce & Bratzke, 2014, 2015, 2017; Bratzke, Bryce, & Seifried-Dübon, 2014; Corallo, Sackur, Dehaene, & Sigman, 2008; Marti, Sackur, Sigman, Dehaene, 2010; for a diverging pattern regarding task switch costs, see Bratzke & Bryce, 2019). To assess people’s introspection, most of these studies used the method of quantified introspection (Corallo et al., 2008), in which participants provide estimates of their RTs (introspective reaction times, IRTs) after each trial. Consistent across several studies, the PRP effect was not reflected in IRTs (Bryce & Bratzke, 2014, 2015; 2017; Corallo et al., 2008; Marti et al., 2010). This result has been interpreted as evidence for a “unified attentional bottleneck” encompassing not only response selection, but also conscious perception (Corallo et al., 2008; Marti et al., 2010; see also Arnell & Jolicoeur, 1999; Marti, Sigman, & Dehaene, 2012; Ruthruff & Pashler, 2001; Tombu et al., 2011).

In light of the intriguing introspective blind spot of the PRP effect, it is noteworthy that introspective abilities for other manipulations of task difficulty are often preserved. For example, Corallo et al. (2008) manipulated the difficulty of perceptual and central processing in a numerical comparison Task 2 (i.e., notation and numerical distance) and observed that both effects were reflected in IRTs. Bryce and Bratzke (2014) used different degrees of stimulus degradation to manipulate perceptual processing in Task 2, and made a similar observation. Thus, so far it seems that the effects of difficulty manipulations, irrespective of whether they are supposed to affect central or perceptual processing, are not subject to the same introspective limitations as the PRP effect.

In the present study, we focused on a particularly interesting case of a difficulty manipulation in Task 1, where compatibility relations between the responses in both tasks affect not only processing of Task 2, but also of Task 1. Such a compatibility-based backward crosstalk effect (BCE) was first demonstrated by Hommel (1998). For example, in Experiment 1 of his study, a colored letter served as the stimulus and participants responded in Task 1 with a manual left or right key press to the color of the letter, and in Task 2 with a vocal “left” or “right” utterance to the identity of the letter. Even RTs in Task 1 (RT1) were shorter in trials with compatible responses (e.g., left key press and vocal “left” utterance) compared with incompatible responses. Similar observations were reported by subsequent studies, some of which extended the result to other responses such as pedal responses in Task 2 (e.g., Ellenbogen & Meiran, 2008; Janczyk, Pfister, Hommel, & Kunde, 2014; Janczyk, Renas, & Durst, 2018; Lien & Proctor, 2000; Miller & Durst, 2014).

A characteristic of the BCE, which is interesting for the present study as well, is its sequential modulation. Such sequential modulations were reported first for the Eriksen flanker task (Eriksen & Eriksen, 1974), where the respective congruency effect is larger following congruent than following incongruent trials (Gratton, Coles, & Donchin, 1992). Similar effects were also reported for other tasks (e.g., Janczyk & Leuthold, 2018; Leuthold & Schröter, 2006; Stürmer, Leuthold, Soetens, Schröter, & Sommer, 2002; Wühr, 2004, 2006). According to a prominent explanation, the cognitive system registers cognitive conflict in incongruent trials, and adjusts subsequent processing to deal with this conflict experience in goal-directed ways (Botvinick, Braver, Barch, Carter, & Cohen, 2001). Similar sequential modulations were also reported for the BCE in several studies (Durst & Janczyk, 2019; Janczyk, 2016; Renas, Durst, & Janczyk, 2018; Scherbaum, Gottschalk, Dshemuchadse, & Fischer, 2015): The BCE is much smaller, sometimes inverted, following incompatible trials compared with following compatible trials. Conceivably, this sequential modulation is an example of a rather subtle effect on RT1s that results from the co-occurrence of several task features. In the present study, we utilize this effect to explore possible limits of introspection regarding difficulty effects observed in Task 1 of a dual task in a trial-by-trial manner.

## Experiment 1

Experiment 1 combined the approach of Janczyk’s (2016) study with the method of quantified introspection. Accordingly, participants performed a dual task with manual responses in Task 1 and pedal responses in Task 2. After each trial, participants were asked to provide IRTs for Task 1 and Task 2.

### Method

#### Participants

Thirty-two students of the University of Tübingen participated for monetary compensation or course credit (mean age = 24.6 years; 19 female). All participants reported normal or corrected-to-normal vision, were naïve regarding the underlying hypotheses, and provided written informed consent prior to data collection.

#### Apparatus and stimuli

Stimulus presentation, response collection, and the experimental procedure were controlled by a standard PC connected to a 17-inch CRT monitor. The responses in Task 1 (R1) were collected with two custom-built response keys placed to the left and right of the participants on the table that were operated with the left and right index finger. The responses in Task 2 (R2) were given on foot switches placed on the floor that were operated with the left and right foot. Task 1 stimuli (S1) were the letters “H” and “S” presented against a black background. The letter appeared first in gray color and changed its color to red or green (the Task 2 stimulus; S2) after an SOA.

#### Tasks and procedure

Participants were instructed to give a manual R1 to the letter identity (S1) and a pedal R2 to the letter color (S2). After both responses were given, they were further asked to provide judgments of their RTs in both tasks using a visual analogue scale. The visual analogue scale consisted of a horizontal line with vertical lines at both ends, which were labeled “0 ms” and “2000 ms”. Three additional vertical lines indicated 500, 1000, and 1500 ms values (without verbal labeling). A left mouse click on the horizontal line placed a red tick on the line. Participants were explicitly instructed (written and oral instruction) to judge the intervals between the onset of the letter and their manual response (RT1) and between the onset of the color change and their pedal response (RT2).

A trial started with the onset of a central white fixation cross (250 ms) when participants pressed both manual response keys simultaneously. Following a blank interval (250 ms), the gray letter appeared (S1), and changed its color (to S2) after an SOA of 50 or 650 ms. Specific error feedback was provided for 1000 ms (e.g., wrong key presses, or unspecific errors such as missing responses within a time limit of 2500 ms or wrong response order), followed by a blank screen (500 ms). In cases without unspecific errors, the visual analogue scale was presented asking participants to judge their RT to S1 (IRT1), and then—following a blank screen of 500 ms—their RT to S2 (IRT2). The next trial started after an inter-trial interval (ITI) of 1000 ms.

Following ten randomly drawn familiarization trials, 10 blocks of 48 trials each were administered; the first two blocks were considered practice and were not analyzed. The 48 trials resulted from 6 repetitions of 2 S1 (H vs. S) × 2 S2 (red vs. green) × 2 SOAs (50 vs. 650 ms). Stimulus–response mappings in both tasks were counterbalanced across participants. Participants were tested individually in one single session of about 90 min. Instructions emphasized speed and accuracy.

#### Design and analyses

A trial was R1–R2 compatible when both responses were to be given on the same side; otherwise, a trial was incompatible. Three independent variables of interest were varied within participants: (1) R1–R2 compatibility in Trial *n* (incompatible vs. compatible), (2) R1–R2 compatibility in Trial *n*−1 (incompatible vs. compatible), and (3) SOA (50 vs. 650 ms). The first trial of each block, and trials with unspecific errors were excluded first (R2 given before R1, R2 given before S2 onset, no response within 2500 ms after S2 onset), and in addition only trials following entirely correct trials were analyzed. Further, only trials with an inter-response interval (IRI) larger than 100 ms were analyzed to exclude an influence of response grouping on the BCE (see Ulrich & Miller, 2008).

For RT and IRT analyses, only trials with both responses correct were considered, and trials were excluded as outliers if either RT1 or RT2 deviated more than 2.5 SDs from the respective cell mean (calculated for each participant separately). Separate ANOVAs with the within-subjects factors SOA, compatibility in Trial *n*, and compatibility in Trial* n*−1 were conducted for error rates (ER1 and ER2), reaction times (RT1 and RT2), and introspective reaction times (IRT1 and IRT2).

### Results

**Fig. 1:**
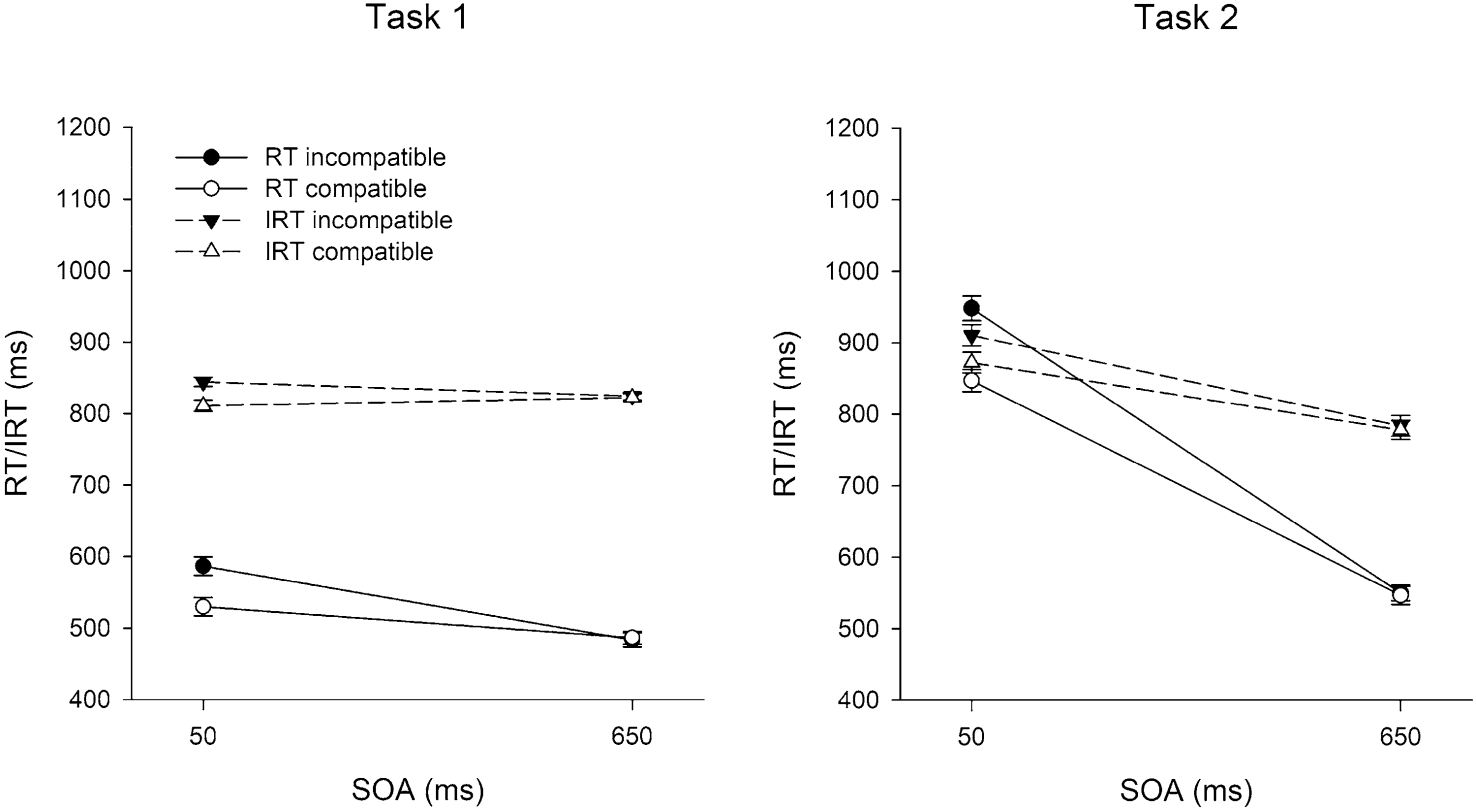
Mean reaction times (RT) and introspective reaction times (IRT) in Task 1 and Task 2 as a function of R1–R2 compatibility and SOA in Experiment 1. Error bars represent ± 1 within-subjects SE

**Fig. 2:**
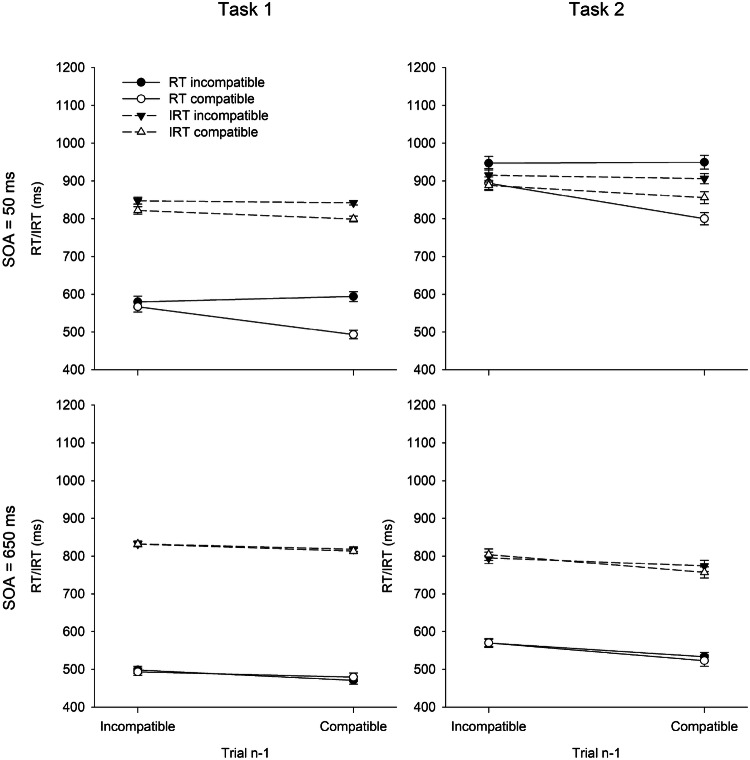
Mean reaction times (RT) and introspective reaction times (IRT) in Task 1 and Task 2 as a function of R1–R2 compatibility in Trial *n*−1, R1–R2 compatibility in Trial *n*, and SOA in Experiment 1. Error bars represent ± 1 within-subjects SE

For RT and IRT analyses, we excluded 4.4% of correct trials as outliers. Figure 1 shows RTs and IRTs in Task 1 and 2 as a function of SOA and R1–R2 compatibility in Trial *n.* The results of the full design, including the sequential modulation of the compatibility effects, are shown in Fig. 2. Within-subjects standard errors were calculated according to Morey (2008). Table 1 summarizes ER1 and ER2.

### Reaction time in Task 1 (RT1)

All main effects were significant. RT1 was longer in short (558 ms) than in long SOA (486 ms) trials, *F*(1, 31) = 20.36, *p* < .001, $\eta_{{\text{p}}}^{2}$ = .40, and there was a BCE of 27 ms, *F*(1, 31) = 11.17, *p* = .002, $\eta_{{\text{p}}}^{2}$ = .26. RT1 was also 25 ms longer after an incompatible than after a compatible trial, *F*(1, 31) = 16.41, *p* < .001, $\eta_{{\text{p}}}^{2}$ = .35. The interaction between SOA and compatibility in Trial *n* was significant, *F*(1, 31) = 13.36, *p* = .001, $\eta_{{\text{p}}}^{2}$ = .30. The BCE was 57 ms at short SOA and virtually nonexistent (− 2 ms) at long SOA. The interaction between SOA and compatibility in Trial *n*−1 was not significant, *F*(1, 31) = 0.98, *p* = .330, $\eta_{{\text{p}}}^{2}$ = .03. However, compatibility in Trial *n* and in Trial *n*−1 showed a significant interaction, *F*(1, 31) = 21.14, *p* < .001, $\eta_{{\text{p}}}^{2}$ = .41. The BCE was larger after compatible (46 ms) than after incompatible (9 ms) trials. Finally, the three-way interaction was also significant, *F*(1, 31) = 16.80, *p* < .001, $\eta_{{\text{p}}}^{2}$ = .35. The sequential modulation of the BCE was much more pronounced at short (101 vs. 13 ms) than at long SOA (5 vs. − 8 ms).

#### Introspective reaction time in Task 1 (IRT1)

In contrast to the objective RT1 pattern, IRT1 was not significantly affected by SOA, *F*(1, 31) = 0.22, *p* = .641, $\eta_{{\text{p}}}^{2}$ = .01. The other two main effects, however, were significant and mirrored the objective RT1 pattern. Accordingly, there was an introspective BCE of 18 ms, *F*(1, 31) = 9.85, *p* = .004, $\eta_{{\text{p}}}^{2}$ = .24, and IRT1 was also 15 ms longer after an incompatible than after a compatible trial, *F*(1, 31) = 11.47, *p* = .002, $\eta_{{\text{p}}}^{2}$ = .27. The introspective BCE was larger at short (34 ms) than at long SOA (3 ms), *F*(1, 31) = 9.66, *p* = .004, $\eta_{{\text{p}}}^{2}$ = .24, again mirroring the objective RT1 pattern. Similarly, there was no significant interaction between SOA and compatibility in Trial *n*−1, *F*(1, 31) = 0.05, *p* = .829, $\eta_{{\text{p}}}^{2}$ < .01. In contrast to the RT1 pattern, however, there was no indication of a sequential modulation of the introspective BCE as a function of compatibility in Trial *n*−1, *F*(1, 31) = 1.97, *p* = .170, $\eta_{{\text{p}}}^{2}$ = .06. Numerically, the introspective BCE was 27 ms after compatible trials, and 13 ms after incompatible trials. The three-way interaction was also not significant, *F*(1, 31) = 0.71, *p* = .406, $\eta_{{\text{p}}}^{2}$ = .02.

#### Task 1 error rate (ER1)

ER1 was not affected by SOA, *F*(1, 31) = 1.14, *p* = .294, $\eta_{{\text{p}}}^{2}$ = .04. Participants made more errors in incompatible (4.0%) than in compatible trials (2.4%), *F*(1, 31) = 9.93, *p* = .004, $\eta_{{\text{p}}}^{2}$ = .24. ER1 was also affected by compatibility in Trial *n*−1, *F*(1, 31) = 5.31, *p* = .028, $\eta_{{\text{p}}}^{2}$ = .15, with more errors after incompatible (3.8%) than after compatible (2.7%) trials. There was a larger BCE at short (5.3 vs. 1.7%) than at long SOA (2.9 vs. 3.1%), *F*(1, 31) = 5.73, *p* = .023, $\eta_{{\text{p}}}^{2}$ = .16. The BCE was also larger after compatible (3.9 vs. 1.3%) than after incompatible (4.1 vs. 3.5%) trials, *F*(1, 31) = 5.37, *p* = .027, $\eta_{{\text{p}}}^{2}$ = .15. The interaction between SOA and compatibility in Trial *n*–1 was also significant, *F*(1, 31) = 5.37, *p* = .027, $\eta_{{\text{p}}}^{2}$ = .15. The effect of compatibility in Trial *n*−1 was smaller at short (3.7 vs. 3.4%) than at long SOA (3.9 vs. 2.1%). Finally, the three-way interaction was also significant, *F*(1, 31) = 4.28, *p* = .047, $\eta_{{\text{p}}}^{2}$ = .12. There was a stronger sequential modulation of the BCE at short than at long SOA (3.4 vs. 0.8 percentage points).

#### Reaction time in Task 2 (RT2)

RT2 showed a clear PRP effect of 348 ms, *F*(1, 31) = 388.23, *p* < .001, $\eta_{{\text{p}}}^{2}$ = .93. There was also a compatibility effect of 54 ms, *F*(1, 31) = 15.54, *p* < .001, $\eta_{{\text{p}}}^{2}$ = .33, and RT2 was 53 ms longer after an incompatible than after a compatible trial, *F*(1, 31) = 15.56, *p* < .001, $\eta_{{\text{p}}}^{2}$ = .33. The interaction between SOA and compatibility in Trial *n* was also significant, *F*(1, 31) = 20.05, *p* < .001, $\eta_{{\text{p}}}^{2}$ = .39, with a compatibility effect of 101 ms at short SOA and of 5 ms at long SOA. The interaction between SOA and compatibility in Trial *n*−1 was not significant, *F*(1, 31) = 0.08, *p* = .780, $\eta_{{\text{p}}}^{2}$ < .01. However, as for RT1, compatibility in Trial *n* and in Trial *n*−1 interacted significantly, *F*(1, 31) = 26.84, *p* < .001, $\eta_{{\text{p}}}^{2}$ = .46. The compatibility effect in Trial *n* was larger after compatible (80 ms) than after incompatible (26 ms) trials. Finally, the three-way interaction was also significant, *F*(1, 31) = 19.38, *p* < .001, $\eta_{{\text{p}}}^{2}$ = .38. The sequential modulation of the compatibility effect was much more pronounced at short (149 vs. 53 ms) than at long SOA (10 vs. 0 ms).

#### Introspective reaction time in Task 2 (IRT2)

There was a clear introspective PRP effect of 110 ms, *F*(1, 31) = 23.26, *p* < .001, $\eta_{{\text{p}}}^{2}$ =.43. IRT2 was also affected by compatibility in Trial *n* (21 ms), *F*(1, 31) = 5.63, *p* = .024, $\eta_{{\text{p}}}^{2}$ = .15, and in Trial *n*−1 (28 ms), *F*(1, 31) = 19.98, *p* < .001, $\eta_{{\text{p}}}^{2}$ = .39. The interaction between SOA and compatibility in Trial *n* was significant, *F*(1, 31) = 7.89, *p* = .009, $\eta_{{\text{p}}}^{2}$ = .20. The introspective compatibility effect was larger at short (38 ms) than at long SOA (4 ms). Numerically, there was also a sequential modulation of the compatibility effect, with a larger compatibility effect after compatible (34 ms) than after incompatible trials (9 ms). However, this interaction did not reach significance, *F*(1, 31) = 3.62, *p* = .066, $\eta_{{\text{p}}}^{2}$ = .10. The interaction between SOA and compatibility and the three-way interaction was not significant, *p*s*>* .243*.*

#### Task 2 error rate (ER2)

ER2 was not affected by SOA, *F*(1, 31) = 0.51, *p* = .480, $\eta_{{\text{p}}}^{2}$ = .02. Participants made more errors in incompatible (7.3%) than in compatible trials (5.2%), *F*(1, 31) = 5.20, *p* = .030, $\eta_{{\text{p}}}^{2}$ = .14, and also more errors after incompatible (7.0%) than after compatible trials (5.4%), *F*(1, 31) = 9.39, *p* = .004, $\eta_{{\text{p}}}^{2}$ = .23. There was a compatibility effect at short SOA (8.1 vs. 3.9%), but no such effect at long SOA (6.5 vs. 6.4%), *F*(1, 31) = 8.64, *p* = .006, $\eta_{{\text{p}}}^{2}$ = .22. Similarly, the compatibility effect was much larger after compatible (7.0 vs. 3.7%) than after incompatible (7.6 vs. 6.6%) trials, *F*(1, 31) = 5.72, *p* = .023, $\eta_{{\text{p}}}^{2}$ = .16. The interaction between SOA and compatibility in Trial *n*−1 and the three-way interaction was not significant, *p*s> .325.

### Discussion

The results showed the standard R1–R2 BCE and its sequential modulation depending on compatibility in Trial *n*−1. Accordingly, a BCE occurred when the two tasks temporally overlapped (i.e., at short SOA), and the effect was larger after compatible than after incompatible trials. Introspective RTs largely reflected the objective pattern, including the PRP effect. This observation is in contrast to the typically observed absence of the PRP effect in IRTs (e.g., Corallo et al., 2008). However, one might argue that the introspective sensitivity was still lower for the PRP effect than for the compatibility effects, as the introspective PRP effect amounted to 31% of the objective effect whereas the introspective compatibility effects amounted to 67% (Task 1) and 40% (Task 2) of the objective effects. Additionally, the PRP effect was not accompanied by the same effect on error rates, so that participants could not infer the PRP effect from their accuracy performance (see Bryce & Bratzke, 2014). Nevertheless, some aspects of the objective result pattern were not reflected in IRT. Specifically, the sequential modulations of the compatibility effects, which appeared in Task 2 as well as in Task 1, were not reflected. Thus, even though introspective abilities were surprisingly good, including the PRP effect, they also showed some limitations regarding the sequential modulation of the compatibility effects.

A comparison of the present results with the same conditions of Experiment 1 in Janczyk (2016) revealed that the sequential modulation of the BCE in Experiment 1 was relatively small (averaged across SOAs: 37 vs. 87 ms). Therefore, we hypothesized that the IRT collection between subsequent trials in Experiment 1 could have disrupted a between-trial sequential adaptation. This suggestion is also in line with previous results showing that sequential modulations of congruency effects rapidly decay when more time elapses between subsequent trials (i.e., with increasing ITI; Duthoo, Abrahamse, Braem, & Notebaert, 2014; Egner, Ely, & Grinband, 2010).

## Experiment 2

Experiment 2 was basically the same as Experiment 1, but we collected IRTs only after each second trial. That is, each trial was essentially a pair of a prime and a test trial, and participants were asked to provide IRTs only for the test trial. With this procedure, we expected a stronger sequential modulation of the BCE than in Experiment 1.

### Method

#### Participants

A new sample of 32 students of the University of Tübingen was recruited for monetary compensation or course credit (mean age = 22.3 years; 24 female). The same criteria as in Experiment 1 were applied.

#### Apparatus, stimuli, tasks, and procedure

In many respect, this experiment was similar to Experiment 1. The main change was that each trial was essentially a pair of a prime and a test trial. The prime trial was exactly as described for Experiment 1, but without assessing IRTs afterward. Instead, after an ITI of 1000 ms, the test trial was presented, which was then followed by the assessment of IRT1 and IRT2 as described for Experiment 1.

Following ten randomly drawn familiarization trials, 12 blocks of 32 trials each were administered; the first two blocks were considered practice and not analyzed. The 32 trials resulted from combining 2 prime-S1 (H vs. S) × 2 prime-S2 (red vs. green) × 2 test-S1 (H vs. S) × 2 test-S2 (red vs. green) × 2 test-SOAs (50 vs. 650 ms). The prime SOA was randomly drawn for each trial. Orthogonally combining all stimuli resulted in equal numbers of trials for each prime trial/test trial transition (i.e., compatible/compatible, incompatible/compatible, compatible/incompatible, incompatible/incompatible).

#### Design and analyses

Analyses followed those described for Experiment 1, but focused on performance in the test trials.

### Results

**Fig. 3:**
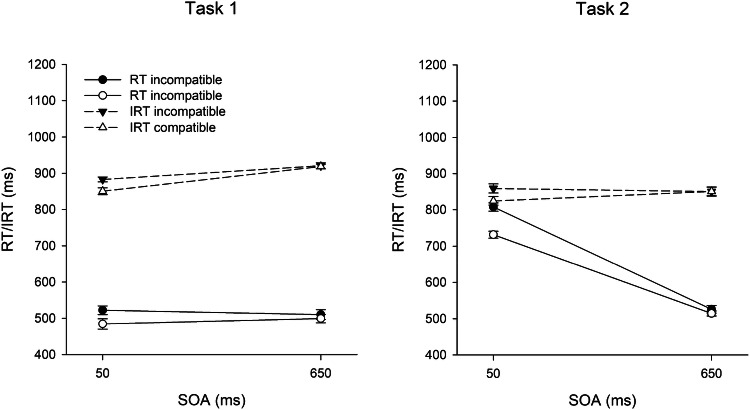
Mean reaction times (RT) and introspective reaction times (IRT) in Task 1 and Task 2 as a function of R1–R2 compatibility and SOA in test trials in Experiment 2. Error bars represent ± 1 within-subjects SE

**Fig. 4:**
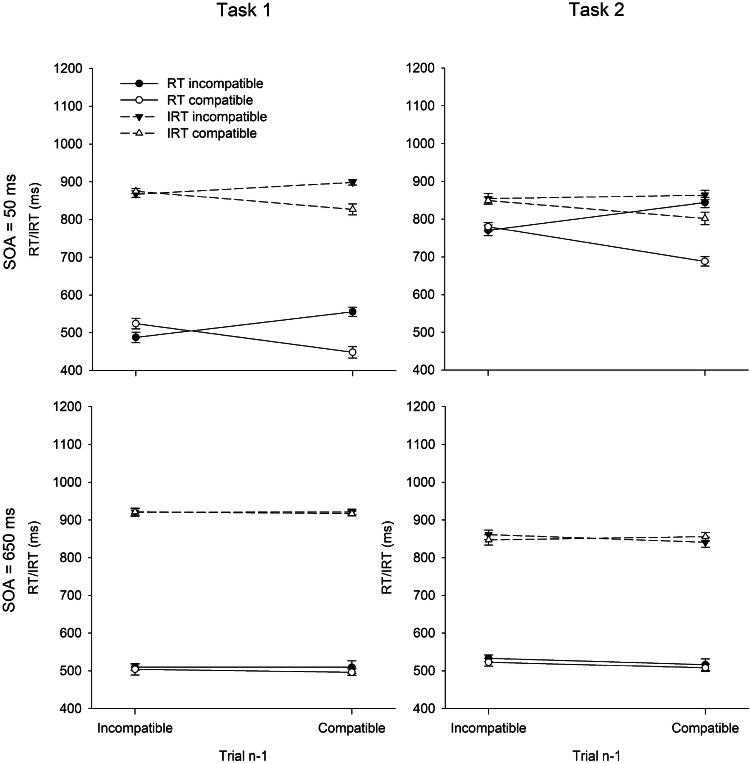
Mean reaction times (RT) and introspective reaction times (IRT) in Task 1 and Task 2 as a function of R1–R2 compatibility in Trial *n*−1, R1–R2 compatibility in Trial *n*, and SOA in test trials in Experiment 2. Error bars represent ± 1 within-subjects SE

For RT and IRT analyses, we excluded 5.0% of correct trials as outliers. Figure 3 shows RTs and IRTs in Task 1 and 2 as a function of SOA and R1–R2 compatibility in Trial *n.* The results of the full design are depicted in Fig. 4. Table 2 summarizes ER1 and ER2.

**Table 1:** Error rates (%) in Task 1 and Task 2 (ER1/ER2) in Experiment 1 as a function of R1–R2 compatibility in Trial *n*–1, R1–R2 compatibility in Trial *n*, and SOA

SOA (ms)	Incompatible Trial *n*−1		Compatible Trial *n*−1	
	Incompatible Trial *n*	Compatible Trial *n*	Incompatible Trial *n*	Compatible Trial *n*
50	4.66/7.98	2.92/4.95	5.77/7.94	0.65/2.74
650	3.70/6.88	4.18/8.11	2.25/6.01	1.94/4.56

**Table 2:** Error rates (%) in Task 1 and Task 2 (ER1/ER2) in test trials in Experiment 2 as a function of R1–R2 compatibility in Trial *n*−1, R1–R2 compatibility in Trial *n*, and SOA

SOA (ms)	Incompatible Trial *n*−1		Compatible Trial *n*−1	
	Incompatible Trial *n*	Compatible Trial *n*	Incompatible Trial *n*	Compatible Trial *n*
50	1.48/4.04	2.03/3.22	7.85/6.68	0.46/1.06
650	1.28/2.68	2.13/4.17	2.04/3.32	0.85/3.15

#### Reaction time in Task 1 (RT1)

In contrast to Experiment 1, there was no SOA effect on RT1, *F*(1, 31) < 0.01, *p* = .963, $\eta_{{\text{p}}}^{2}$ < .01. There was again a BCE of 22 ms, *F*(1, 31) = 25.11, *p* < .001, $\eta_{{\text{p}}}^{2}$ = .45. However, RT1 was not significantly affected by compatibility in Trial *n*−1, *F*(1, 31) = 0.63, *p* = .432, $\eta_{{\text{p}}}^{2}$ = .02. The interaction between SOA and compatibility in Trial *n* again reached significance, *F*(1, 31) = 4.36, *p* = .045, $\eta_{{\text{p}}}^{2}$ = .12. The BCE was larger at short (35 ms) than at long SOA (10 ms). As in Experiment 1, the interaction between SOA and compatibility in Trial *n*−1 was not significant, *F*(1, 31) < 0.01, *p* = .989, $\eta_{{\text{p}}}^{2}$ < .01, but the sequential modulation of the compatibility effect and the three-way interaction were significant. The BCE was 60 ms after compatible trials and slightly reversed (− 15 ms) after incompatible trials, *F*(1, 31) = 21.02, *p* < .001, $\eta_{{\text{p}}}^{2}$ = .40, and this modulation of the BCE was much more pronounced at short (107 vs. − 37 ms) than at long SOA (13 vs. 6 ms), *F*(1, 31) = 34.36, *p* < .001, $\eta_{{\text{p}}}^{2}$ = .53.^1^

#### Introspective reaction time in Task 1 (IRT1)

In contrast to the objective RT1 pattern, IRT1 was significantly affected by SOA, *F*(1, 31) = 18.82, *p* < .001, $\eta_{{\text{p}}}^{2}$ = .38. IRT1 was 53 ms shorter at short than at long SOA. Regarding the BCE, IRT1s again reflected the objective pattern, *F*(1, 31) = 9.85, *p* = .004, $\eta_{{\text{p}}}^{2}$ = .24, with an introspective BCE of 17 ms. Also in line with the objective RT1 pattern, IRT1 was not affected by compatibility in Trial *n*−1, *F*(1, 31) = 0.80, *p* = .379, $\eta_{{\text{p}}}^{2}$ = .03. The two-way interaction pattern again reflected the objective RT1 pattern. Accordingly, there were significant interactions between SOA and compatibility in Trial *n*, *F*(1, 31) = 14.66, *p* = .001, $\eta_{{\text{p}}}^{2}$ = .32, and between compatibility in Trial *n* and Trial *n*−1, *F*(1, 31) = 18.53, *p* < .001, $\eta_{{\text{p}}}^{2}$ = .37, whereas the interaction between SOA and compatibility in Trial *n*−1 was not significant, *F*(1, 31) = 0.80, *p* = .377, $\eta_{{\text{p}}}^{2}$ = .03. Specifically, participants reported a BCE at short (32 ms) but not at long SOA (2 ms), and also after a compatible (38 ms), but not after an incompatible (− 4 ms) trial. Finally, the significant three-way interaction confirmed that the sequential modulation of the introspective BCE was stronger at short SOA (72 vs. − 8 ms) than at long SOA (4 ms vs. − 1 ms), *F*(1, 31) = 9.23, *p* = .005, $\eta_{{\text{p}}}^{2}$ = .23.

#### Task 1 error rate (ER1)

In contrast to Experiment 1, participants made more errors in short (3.0%) than in long SOA (1.5%) trials, *F*(1, 31) = 8.35, *p* = .007, $\eta_{{\text{p}}}^{2}$ = .21. They also made more errors in incompatible (3.2%) than in compatible (1.3%) trials, *F*(1, 31) = 11.38, *p* = .002, $\eta_{{\text{p}}}^{2}$ = .27. ER1 was also affected by compatibility in Trial *n*−1, *F*(1, 31) = 11.67, *p* = .002, $\eta_{{\text{p}}}^{2}$ = .27, with less errors after incompatible (1.7%) than after compatible (2.8%) trials. The BCE was larger at short (4.9 vs. 1.2%) than at long SOA (1.7 vs. 1.4%), *F*(1, 31) = 11.85, *p* = .002, $\eta_{{\text{p}}}^{2}$ = .28. The interaction between SOA and compatibility in Trial *n*−1 was also significant (short SOA: 1.7 vs. 4.2%; long SOA: 1.7 vs. 1.4%; incompatible vs. compatible, respectively), *F*(1, 31) = 10.88, *p* = .002, $\eta_{{\text{p}}}^{2}$ = .26. There was also a sequential modulation of the BCE due to compatibility in Trial *n*−1, *F*(1, 31) = 20.50, *p* < .001, $\eta_{{\text{p}}}^{2}$ = .40. The BCE was larger after compatible (4.9 vs. 0.7%) than after incompatible trials (1.4 vs. 2.1%). Finally, the three-way interaction was also significant, *F*(1, 31) = 15.45, *p* < .001, $\eta_{{\text{p}}}^{2}$ = .33. There was a stronger sequential modulation of the BCE at short than at long SOA (7.9 vs. 2.9 percentage points).

#### Reaction time in Task 2 (RT2)

RT2 again showed a clear PRP effect of 348 ms, *F*(1, 31) = 358.66, *p* < .001, $\eta_{{\text{p}}}^{2}$ = .92. There was also again a compatibility effect of 41 ms, *F*(1, 31) = 32.92, *p* < .001, $\eta_{{\text{p}}}^{2}$ = .33. However, RT2 was not significantly affected by compatibility in Trial *n*−1, *F*(1, 31) = 2.89, *p* = .099, $\eta_{{\text{p}}}^{2}$ = .09. The interaction pattern was very similar to Experiment 1. Accordingly, the interaction between SOA and compatibility in Trial *n* was significant, *F*(1, 31) = 19.58, *p* < .001, $\eta_{{\text{p}}}^{2}$ = .39, with a compatibility effect of 73 ms at short SOA and of 10 ms at long SOA. The interaction between SOA and compatibility in Trial *n*–1 was not significant, *F*(1, 31) = 0.41, *p* = .526, $\eta_{{\text{p}}}^{2}$ = .01, but compatibility in Trial *n* and in Trial *n*−1 interacted significantly, *F*(1, 31) = 14.99, *p* = .001, $\eta_{{\text{p}}}^{2}$ = .33. The compatibility effect in Trial *n* appeared after compatible (82 ms) but not after incompatible (1 ms) trials. Finally, the three-way interaction was also significant, *F*(1, 31) = 34.67, *p* < .001, $\eta_{{\text{p}}}^{2}$ = .53. There was a clear sequential modulation of the compatibility effect at short (156 vs. −10 ms) but not at long SOA (8 vs. 11 ms).^2^

#### Introspective reaction time in Task 2 (IRT2)

In contrast to Experiment 1, there was no effect of SOA on IRT2, *F*(1, 31) = 0.18, *p* = .676, $\eta_{{\text{p}}}^{2}$ = .01. However, IRT2 was again affected by compatibility in Trial *n* (16 ms), *F*(1, 31) = 8.60, *p* = .006, $\eta_{{\text{p}}}^{2}$ = .22. IRT2 was also again longer after incompatible (853 ms) than after compatible (840 ms) trials. This effect, however, did not reach significance, *F*(1, 31) = 3.64, *p* = .066, $\eta_{{\text{p}}}^{2}$ = .10. In contrast to Experiment 1, the interaction between SOA and compatibility in Trial *n* was significant, *F*(1, 31) = 14.75, *p* = .001, $\eta_{{\text{p}}}^{2}$ = .32. As in objective RT2 performance, there was a compatibility effect at short (33 ms) but not at long SOA (0 ms). Numerically, there was also again a sequential modulation of the compatibility effect, with a larger compatibility effect after compatible (24 ms) than after incompatible trials (9 ms). This interaction, however, again did not reach significance, *F*(1, 31) = 1.23, *p* = .277, $\eta_{{\text{p}}}^{2}$ = .04. In contrast to Experiment 1, the three-way interaction was significant, *F*(1, 31) = 11.79, *p* = .002, $\eta_{{\text{p}}}^{2}$ = .28*.* Similar to the objective RT2 pattern, the sequential modulation of the introspective compatibility effect was stronger at short (62 vs. 5 ms) than at long SOA (− 15 vs. 14 ms).

#### Task 2 error rate (ER2)

As in Experiment 1, ER2 was not affected by SOA, *F*(1, 31) = 0.69, *p* = .412, $\eta_{{\text{p}}}^{2}$ = .02. Participants made more errors in incompatible (4.2%) than in compatible trials (2.9%), *F*(1, 31) = 7.57, *p* = .010, $\eta_{{\text{p}}}^{2}$ = .20. However, there was no significant effect of compatibility in Trial *n*−1 on ER2, *F*(1, 31) = 0.01, *p* = .940, $\eta_{{\text{p}}}^{2}$ < .01. There was a compatibility effect at short (5.5 vs. 2.1%) but not at long SOA (3.0 vs. 3.6%), *F*(1, 31) = 6.01, *p* = .020, $\eta_{{\text{p}}}^{2}$ = .16. The compatibility effect was larger after compatible (4.9 vs. 2.2%) than after incompatible (3.3 vs. 3.8%) trials, *F*(1, 31) = 6.67, *p* = .015, $\eta_{{\text{p}}}^{2}$ = .18. The interaction between SOA and compatibility in Trial *n*−1 was not significant, *F*(1, 31) = 0.36, *p* = .553, $\eta_{{\text{p}}}^{2}$ < .01. However, the three-way interaction was significant, *F*(1, 31) = 5.95, *p* = .021, $\eta_{{\text{p}}}^{2}$ = .16, indicating that the sequential modulation of the compatibility effect was stronger at short than at long SOA (4.8 vs. 1.7 percentage points).

### Discussion

As we expected, the pairwise presentation of prime and test trials in Experiment 2 resulted in a stronger sequential modulation of the BCE than in Experiment 1 (averaged across SOAs: 75 vs. 37 ms). As another result, participants now reported in their IRTs not only the BCE but also its sequential modulation. Additionally, a similar sequential modulation of the compatibility effect in Task 2 was observed, and this modulation was also numerically reflected in IRTs. Experiment 2 exhibited a different picture regarding introspection about the PRP effect, as participants did not report an introspective PRP effect. Overall, participants were remarkably sensitive to the relatively subtle effects of R1–R2 compatibility and their sequential modulations while being blind to the dominating PRP effect.

## General discussion

The present experiments replicate the standard BCE and its sequential modulation in dual-task performance. Accordingly, response compatibility between the two tasks affected not only Task 2 but also Task 1 performance, and this effect was stronger after compatible than after incompatible trials. Our main interest was how participants would introspect about the objective result pattern in this quite complex dual-task situation. Regarding the BCE and its sequential modulation, introspective RTs largely reflected the objective pattern. This was especially the case in Experiment 2 with the pairwise trial procedure, where participants reported in their IRTs not only the BCE but also the SOA-dependent sequential modulation of the BCE. Remarkably, in Experiment 1, the PRP effect was reflected in IRTs. The results of Experiment 2, however, showed the typically observed absence of the PRP effect in IRTs. Thus, Experiment 1 appears to be one of the rare exceptions in which participants report a PRP effect in their IRTs (see also Experiment 2 of Bryce & Bratzke, 2014).

Another interesting aspect of the present results is the apparent longevity of the sequential modulation of the BCE. Previous results on sequential modulations in other conflict tasks have shown that these effects rapidly decay when more time elapses between subsequent trials (Duthoo et al., 2014; Egner et al., 2010). Note that in Experiment 1 of the present study, there was not only a prolongation of the ITI, but participants had to indicate their IRTs during this interval, a procedure that usually takes up to a few seconds. Importantly, we still observed a significant sequential modulation of the BCE, even though the effect was smaller than in Experiment 2, where no additional delay due to the assessment of IRTs occurred. This longevity might be conceived as a problem for a conceptualization of the sequential modulation of the BCE as conflict adaptation (see, e.g., Janczyk, 2016). However, it remains an open question if and how the assessment of IRTs plays a crucial role in the persistence of the sequential modulation over time. It is, for example, conceivable that the demand to provide IRTs, which may require maintaining a short-term representation of RTs, strengthens a possible experience of conflict, which in turn counteracts the dissipation of the sequential modulation.

Previous conceptualization of IRTs in dual tasks in light of the unified attentional bottleneck model imply that IRTs reflect relatively veridical time estimates of the consciously accessible internal processing times (Corallo et al., 2008; Marti et al., 2010). Others have proposed that introspective RTs in dual-task situations reflect retrospective inferences based on a variety of cues (Bratzke & Bryce, 2016, 2019; Bratzke et al., 2014; Bryce & Bratzke, 2014, 2017; see also Klein & Stolz, 2018). In line with the latter assumption, one could argue that in the present study, participants inferred their IRTs from, for example, their experience of the between-trial sequence of R1–R2 compatibility or of the sequence of required and performed responses (e.g., 50% of compatible–compatible and incompatible–incompatible sequences include full response repetitions). It is unlikely, however, that participants inferred the BCE from these experiences, because then they should have been insensitive to the SOA dependency of the BCE and its sequential modulation.

Other potential sources of information for IRTs may be the feeling of difficulty (Bryce & Bratzke, 2014) and the feeling of conflict (or the “urge-to-err”, e.g., Morsella, Wilson, Berger, Honhongva, Gazzaley, & Bargh, 2009; Questienne, Atas, Burle, & Gevers, 2018). Even though these subjective aspects of cognitive control are certainly highly related, if not indistinguishable (but see Questienne, van Dijck, & Gevers, 2018), they have been differently conceptualized. In an introspective PRP study, Bryce and Bratzke (2014) observed a close relationship between IRTs and the feeling of difficulty, and a better match of these variables with error rates than with objective RTs. From these results, the authors concluded that IRTs are strongly influenced by the feeling of difficulty and that error performance can serve as a proxy for the subjective feeling. In the present experiments, error rates largely mirrored RT performance, with the exception of the PRP effect. An exclusive use of this information, however, seems also unlikely because participants did not reliably indicate a sequential modulation of the BCE in Experiment 1, although both error rates as well as RTs showed such a modulation. Additionally, in Experiment 1, participants reported a PRP effect in their IRTs although there was no such effect on error rate.

In the standard PRP paradigm, potential input and output conflicts are usually minimized to investigate the “pure” central (or attentional) costs of dual tasking (e.g., Pashler, 1994). In this situation, the rather unspecific feeling of difficulty may be an appropriate aspect of subjective experience to ask for. In conflict tasks (as, e.g., the BCE task in the present study, or the flanker, the Simon, and the Stroop Task), however, the more specific feeling of conflict (or the “urge-to-err”) may better describe the participants’ subjective experience of their performance. Based on our intuitive understanding of conflict tasks, the feeling of conflict and objective RTs should be highly related. A recent study by Questienne, Atas et al. (2018) indeed provided evidence for such a relationship between RT and the urge-to-err in a simple conflict task (masked priming task). A second important variable related to the urge-of-err in this study was response competition (indexed by EMG activity on the wrong response hand), which modulated the relationship between RT and the urge-to-err, with a much steeper slope of the RT‒urge-to-err function in case of apparent response conflict. Bratzke and Bryce (2019) observed a similar linear relationship between objective and introspective RT in an introspective task-switching study. Consequently, IRTs and the feeling of conflict are probably highly related. The precise relationship of these two measures, however, can only be speculated upon; that they simply reflect confounded experiences seems to be just as possible as that one of them forms the basis for the other.

It is conceivable that the experience of conflict still plays an important role in the awareness of the BCE. According to the influential conflict monitoring theory by Botvinick et al. (e.g., Botvinick et al., 2001), the anterior cingulate cortex (ACC) monitors conflict and triggers cognitive control adjustments in case of conflict detection. Even though this theory does not make any explicit assumptions about the awareness or unawareness of conflict detection and the subsequent control adjustments, these internal signals are probably accessible to introspection (for the role of ACC activity in consciousness, see, e.g., Dehaene et al., 2004; Mayr, 2004; Qin et al., 2010). This consideration is also of interest with respect to the dissociation between the unawareness of the PRP effect in the present and previous studies, and the awareness of switch costs in Bratzke and Bryce (2019) and compatibility (or conflict) effects in the present study. Activation of the ACC has been consistently reported for task switching (e.g., Braver, Reynolds, Donaldson, & Louis, 2003; Dove, Pollmann, Schubert, Wiggins, & von Cramon, 2000; Hyafil, Summerfield, & Koechlin, 2009) and conflict tasks (see, e.g., Botvinick et al., 2001). However, the brain regions specifically involved in dual tasking are less clear (see Wu, Liu, Hallett, Zheng, & Chan, 2014), especially regarding the PRP paradigm (e.g., Dux, Ivanoff, & Asplund, 2006; Jiang, Saxe, & Kanwisher, 2004; Szameitat, Schubert, Mu, & von Cramon, 2002). For example, a study by Jiang et al. (2004) observed no engagement of the ACC or any other brain regions usually associated with cognitive control, when they contrasted a short with a long SOA condition in the PRP paradigm. Overall, the pattern of ACC activation associated with these effects seems to match with the dissociation regarding awareness of the effects in introspective RT studies. Based on these considerations, one can speculate that conflict detection by the ACC and/or subsequent cognitive control adjustments play a role in the awareness of effects like switch costs or the BCE. However, that a non-engagement of the ACC can explain the unawareness of the PRP effect appears very unlikely to us, as the effects of other difficulty manipulations usually not associated with ACC activation or conflict adaptation (e.g., numerical distance, stimulus degradation) have been reflected in IRTs (Bryce & Bratzke, 2014; Corallo et al., 2008).

More important, in terms of mental stage models the dissociation between the unawareness of the PRP effect and the awareness of the BCE and switch costs can be explained by a passive postponement of the central processing stage in case of the PRP effect, and a prolongation of the central stage due to active task preparation in task switching (see Bratzke & Bryce, 2019) and to conflict resolution in case of the BCE. This explanation thus combines assumptions about the processing dynamics in task switching and conflict task performance with the basic assumptions of the unified bottleneck model (Corallo et al., 2008; Marti et al., 2010; Tombu et al., 2011). What are the implications of this explanation for theorizing about the nature of the BCE? According to the conscious perception bottleneck model, conscious perception of Task 2 is delayed as long as the central processor is engaged in response selection for Task 1 (Corallo et al., 2008; Marti et al., 2010). Additionally, conscious access in dual tasks seems to be restricted to central processing (see Corallo et al., 2008; Marti et al., 2010). To reconcile the BCE with traditional bottleneck models, a sub-division of the central stage was suggested (Hommel, 1998; Lien & Proctor, 2002; see also Schubert, Fischer, & Stelzel, 2008). According to this idea, a first stage of response activation runs in parallel with other stages of simultaneous tasks, while only the second stage of (final) response selection is considered a bottleneck process. With sufficient temporal overlap between response activations, mutual crosstalk between the two tasks can occur. More recent studies, however, preferred a model where some automatic Task 2 response activation occurs, but directly affects the capacity-limited stage of response selection in Task 1 (Durst & Janczyk, 2019; Janczyk et al., 2018; Thomson, Danis, & Watter, 2015). While the response activation account suggests a postponement of Task 1 response selection, the latter account suggests that the duration of Task 1 response selection proper varies.

If one follows the assumption that in dual tasks conscious access is restricted to central processing, the present awareness of the BCE and its sequential modulation would indicate that the BCE arises from a prolongation rather than from a postponement of Task 1 response selection. This is line with the assumption that Task 2 response activation directly affects the duration of Task 1 response selection (Janczyk et al., 2018; Thomson et al., 2015). It is important to note, however, that previous results by Bryce and Bratzke (2014) argue against the”exclusive central access” assumption, at least for Task 1. In this study, the effect of stimulus degradation in Task 1 was reflected in IRTs, suggesting that perceptual (or pre-central) processing in Task 1 can be consciously accessible. Under this assumption, the present results would be consistent with both a locus of the BCE in Task 1 response activation as well as in Task 1 response selection.

Since the discovery of sequential conflict-modulation effects in the 90s of the last century (Gratton et al., 1992), there has been a discourse on the role of consciousness in conflict adaptation (e.g., Desender & Van den Bussche, 2012; Kunde, Reuss, & Kiesel, 2012; Mayr, 2004). Recent evidence suggests that conflict awareness plays an important role in conflict adaptation (Ansorge, Fuchs, Khalid, & Kunde, 2011; Desender, Van Opstal, & Van den Bussche, 2014; Fröber, Stürmer, Frömer, & Dreisbach, 2017, Kunde, 2003; Questienne, Van Opstal, van Dijck, & Gevers, 2018). Some of these studies used masking to manipulate the awareness of conflict information (Ansorge et al., 2011; Kunde, 2003), whereas others directly asked for ratings of conflict (Desender et al., 2014) or pleasantness (Fröber et al., 2017). The present result that the sequential modulation of the BCE was accompanied by an introspective trial-by-trial awareness of congruency effects on objective RT is certainly consistent with the suggestion of these studies. However, whether awareness of RT effects is crucial for the sequential conflict adaptation or rather an epiphenomenon remains an open question for future research.

In conclusion, the present study investigated participants’ ability to introspect about the effect of between-task crosstalk in dual tasks: while participants are typically unaware of the dominating PRP effect, here they showed awareness of the SOA-dependent sequential modulation of the BCE. This demonstrates that people’s introspection about the temporal processing demands in a rather complex dual-task situation is intriguingly accurate, but also severely limited at the same time.

## Footnotes

1 We also performed an ANOVA with the additional factor trial type (prime vs. test trial) on RT1. Concerning this factor, the analysis revealed a significant main effect, a significant two-way interaction with SOA, significant three-way interactions with SOA and compatibility in Trial *n*−1, and with compatibility in Trial *n* and compatibility in Trial *n*−1, and a significant four-way interaction, *p*s < .033. In comparison with test trials, RT1s in prime trials were on average longer (538 vs. 504 ms), and the sequential modulation of the BCE was less pronounced (short SOA: 115 vs. 52 ms; long SOA: 2 vs. − 4 ms).

2 We also performed an ANOVA with the additional factor trial type (prime vs. test trial) on RT2. Concerning this factor, the analysis revealed a significant main effect, a significant two-way interaction with SOA, and a significant four-way interaction, *p*s < .040. Similar to the RT1 pattern, RT2 was on average longer (748 vs. 645 ms), and the sequential modulation of the BCE less pronounced (short SOA: 193 vs. 85 ms; long SOA: 57 vs. 26 ms) in prime compared with test trials.
